# Influence of simulated microgravity on the activation of the small GTPase Rho involved in cytoskeletal formation – molecular cloning and sequencing of bovine leukemia-associated guanine nucleotide exchange factor

**DOI:** 10.1186/1471-2091-7-19

**Published:** 2006-06-28

**Authors:** Akira Higashibata, Mari Imamizo-Sato, Masaya Seki, Takashi Yamazaki, Noriaki Ishioka

**Affiliations:** 1 Institute of Space and Astronautical Science, Japan Aerospace Exploration Agency, Tsukuba Space Center, 2-1-1 Sengen, Tsukuba, Ibaraki 305-8505, Japan; 2 Department of Space Environmental Medicine, Kagoshima University Graduate School of Medical and Dental Science, 8-35-1, Sakuragaoka, Kagoshima, Kagoshima, 890-8544, Japan; 3 Space Station Engineering Department, Advanced Engineering Services Co., Ltd., Tsukuba Mitsui Building, 1-6-1, Takezono, Tsukuba, Ibaraki 305-0032, Japan

## Abstract

**Background:**

The irregular formation of cytoskeletal fibers in spaceflown experimental cells has been observed, but the disorganization process of fibers is still poorly understood. It is well known that the activation of the small GTPase Rho leads to actin stress fibers assembly. This study was performed to evaluate the effect of simulated microgravity on the activation of Rho that is involved in actin fiber remodeling in cells.

**Results:**

Clinorotation influences actin fiber remodeling and its related signaling pathways that involve the small GTPase Rho. Actin stress fiber remodeling was significantly inhibited to a greater extent in cells cultured under clinorotation than in static cultured cells. From the gene and protein expression analyses, we found that the expression level of leukemia-associated Rho guanine nucleotide exchange factor (LARG), which activates Rho, was downregulated under clinorotation. Moreover, we identified the full-length LARG cDNA. The amount of GTP-bound RhoA, that is, the active form of RhoA, decreased under this condition.

**Conclusion:**

The activation of the small GTPase Rho was influenced by simulated microgravity generated by a three-dimensional (3D) clinostat. Furthermore, the full-length cDNA of bovine LARG, a member of the Rho guanine nucleotide exchange factor (GEF) family, was identified, and its gene expression was observed to be downregulated under clinorotation. This downregulation subsequently resulted in the repression of RhoA activation. These results indicated that the disorganization of the actin fibers was caused by the inhibition of Rho activation by 3D clinorotation.

## Background

Gravity is a universal force and affects everything in nature. Gravitational effects on biological events and functions, including the mechanism of gravity sensation in cells, remain unclear. Previous spaceflight experiments have reported the irregular formation of cytoskeletal fibers in spaceflown experimental cells. Lewis *et al*. observed that the microtubule filaments extended from a poorly defined centrosome in human lymphocytes (Jurkat cells) [1]. Gruener and Hughes-Fulford reported that actin reorganization responded to the gravity level and showed abnormal assembly of actin stress fibers [2,3]. In the field of space biology, to examine the effects of microgravity on biological events in ground-based research, clinostat is often used to simulate the microgravity condition. A three-dimensional (3D) clinostat is an apparatus that nullifies the effect of gravity, and it has been used in substitution studies on microgravity effects [2,4-9]. In a study on *Xenopus *myocytes, 3D clinorotation caused disorganization, condensation, and irregular arrangement of the actin filaments [10].

The Rho family of GTPases plays an important role in controlling the organization and remodeling of the actin-based cytoskeleton, and these GTPases act as molecular switches that are active in the GTP-bound state and inactive in the unbound state [11-14]. The activation of Rho is catalyzed by the Dbl family of guanine nucleotide exchange factors (GEFs) [15,16], and activated Rho proteins subsequently induce the formation of actin stress fibers in mammalian cells [12]. The Dbl family comprises a number of proteins with variable modular structures, expression patterns, and cellular functions [17,18]. Leukemia-associated RhoGEF (LARG) is one of the RhoA-selective RhoGEFs that are directly regulated by activated G_α12/13 _proteins and play a key role in oncogenic transformation induced by G protein-coupled receptors [19-21].

In this study, we found that the activation of the small GTPase Rho was influenced by the simulated microgravity generated by the 3D clinostat. Furthermore, the full-length cDNA of bovine LARG was identified, and its gene expression was downregulated under clinorotation. This downregulation subsequently resulted in the repression of RhoA activation. These results indicated that the disorganization of the actin fibers was caused by the inhibition of Rho activation by 3D clinorotation.

## Results

### Formation of actin filaments under clinorotation

**Figure 1:**
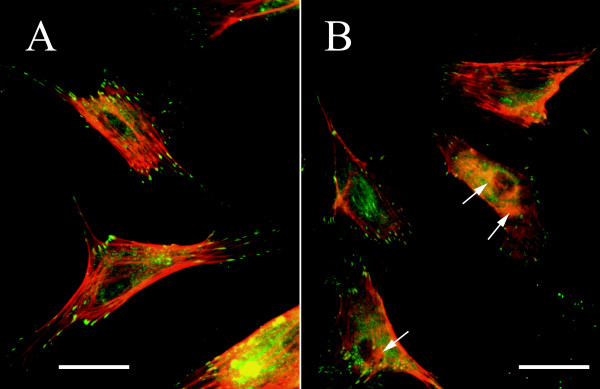
**Effect of altered gravity on actin stress fiber formation in BBME cells**. Panels A and B indicate the control and clinorotated cells, respectively. Clinorotation (B) results in the disorganization of actin stress fibers. BBME cells were cultured as described in the Methods section. After 72 h, the cells were fixed and stained with rhodamine-phalloidin and anti-paxillin antibody in combination with Alexa Fluor 488 to detect the actin stress fibers and paxillins, respectively. The cells were observed using a fluorescence microscope at ×400 magnification. The panels in the figures represent the best estimate of a typical field of cells under each condition. Scale bar = 10 μm.

Disorganization of the actin cytoskeleton has been demonstrated in previous studies on spaceflight and ground-based simulation of microgravity. We determined whether the disorganization of the actin cytoskeleton is observed in the bovine brain microvascular endothelial (BBME) cells cultured in the simulated microgravity condition. We first observed the F-actin filament by rhodamine-phalloidin staining. BBME cells are adherent cells and normally show actin stress fibers (red) under the stationary condition as shown in Figure 1A. After exposure to clinorotation for 72 h, the cores of the actin filaments formed clusters, which are indicated by arrows in Figure 1B, and stress fibers were distinctly observed in the control cells. Paxillins (green), which are cytoskeletal proteins involved in actin-membrane attachment, were also observed more distinctly in the control cells than in the clinorotated cells. These observations clearly indicated that clinorotation caused the disorganization of the actin cytoskeleton.

### Reverse transcription polymerase chain reaction-based differential display, cloning, and sequencing of bovine LARG

In order to study the effect of altered gravity on gene expression in the cells, reverse transcription polymerase chain reaction (RT-PCR)-based differential display, which is an mRNA fingerprinting technique, was performed. RT-PCR was performed using the total RNA isolated from the clinorotated and stationary control cells as templates and all the 60 primer sets listed in the additional files (see Additional Files Table 1). Therefore, a total of 60 PCRs were run, and each contained a different combination of 5' and 3' primers. From the polyacrylamide gel electrophoresis images, we identified 62 DNA segments that showed different expression levels under clinorotation (data not shown). The DNA sequences of some segments could be determined, and homology searches were performed using the Basic Local Alignment Search Tool (BLAST) program from the DNA Data Bank of Japan (DDBJ), Shizuoka. Although the function of the identified segments was unknown, two segments showed high homology to human RhoGEF or the activated leukocyte cell adhesion molecule.

**Figure 2:**
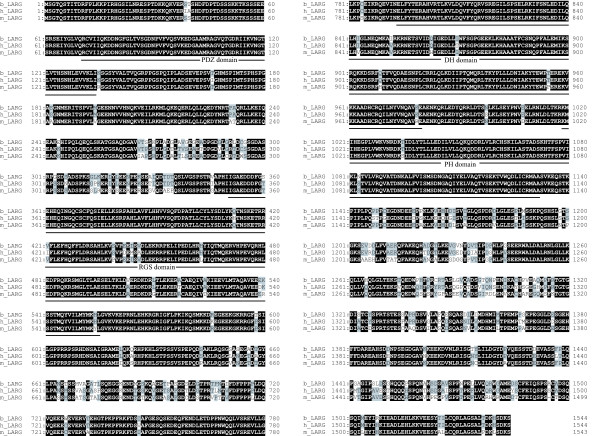
**Sequence alignment of the bovine, human, and mouse LARG proteins**. Identical amino acids are indicated by the white characters on the black background, and partially identical residues are indicated by the gray background. The locations of the PDZ, RGS, PH, and DH domains are indicated by lines.

One of the segments that showed differential expression levels in the mRNA fingerprinting demonstrated substantial similarity to the 3' region of human LARG mRNA, which is one of the RhoGEFs. We performed several cloning experiments to obtain longer cDNAs corresponding to this segment. Full-length bovine LARG cDNA, including the open reading frame (ORF), was obtained using the RT-PCR, 5' rapid amplification of cDNA ends (RACE), and 3' RACE methods. The resulting assembled cDNA comprised 8,364 nucleotides including a 4,635-bp ORF corresponding to a predicted 1,544 amino acid (aa) polypeptide similar to that of human LARG. The predicted protein showed the greatest similarity to human and mouse LARG proteins (92.3% and 89.4% aa identity, respectively). The molecular weight of the protein was estimated as 172.7 kDa; this is the same as that of the human and mouse LARG proteins. Therefore, we concluded that this protein was bovine LARG. The alignments of the bovine, human, and mouse protein sequences are shown in Figure 2 (the nucleotide sequence of bovine LARG cDNA will appear in the DDBJ/EMBL/GenBank database [DDBJ/EMBL/GenBank: AB188499]). No other protein sequence in GenBank, except the bovine LARG and translated bovine LARG expressed sequence tags, showed a higher similarity to the human and mouse LARG proteins. These facts strongly indicate that the cloned bovine LARG cDNA corresponds to a bonafide bovine orthologue of the human gene. Motif analysis of the bovine LARG protein sequence revealed the presence of four conserved functional domains, namely, the closely juxtaposed Dbl homology (DH) and pleckstrin homology (PH) domains, the PDZ domain and the regulator of G protein signaling (RGS) domain. As expected, the PDZ and PH domains of the bovine, human, and mouse proteins are highly conserved, and they showed the highest degree of sequence conservation. The PDZ domains of these proteins are identical, whereas the PH domains differ by a single conservative amino acid substitution. It is important to note the remarkably high degree of conservation of the entire N-terminal part of the LARG protein encompassing the PDZ domain. Within the first 265 amino acid residues, substitutions were observed in several amino acids, indicating an overall identity of 98%. In comparison, the regions between the PDZ and RGS domains and the RGS and DH domains as well as the entire C-terminal domains are substantially more variable. For example, the region between the PH domain and the C terminal of bovine LARG has an aa identity of 84.5% and 79.9% with those of human and mouse LARG, respectively.

### Real-time quantitative PCR

**Figure 3:**
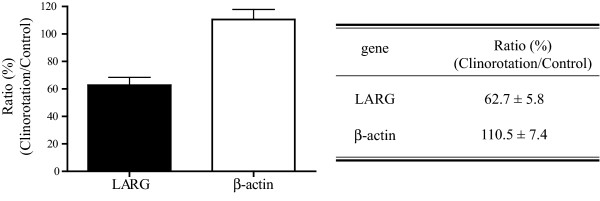
**Real-time quantitative PCR**. The expression of LARG mRNA was measured by real-time quantitative PCR as described in the Methods section. The LARG gene expression in the clinorotated cells decreased to 57.5% of that in the stationary control cells. Values were obtained from seven individual experiments in triplicates and are represented as means ± SE (*p *< 0.05).

From the mRNA fingerprinting results, we predicted that the LARG gene expression was downregulated under clinorotation. To confirm the change in the LARG gene expression under clinorotation, real-time quantitative PCR was performed. As shown in Figure 3, the level of LARG gene expression in the clinorotated cells decreased to 62.7 ± 5.8% of that in the stationary control cells. This result indicated that under clinorotation, the LARG gene expression was downregulated at the mRNA level, and it suggested that Rho activation would be influenced by changes in the gravity level.

### Western blot analysis and Rho activation assay

**Figure 4:**
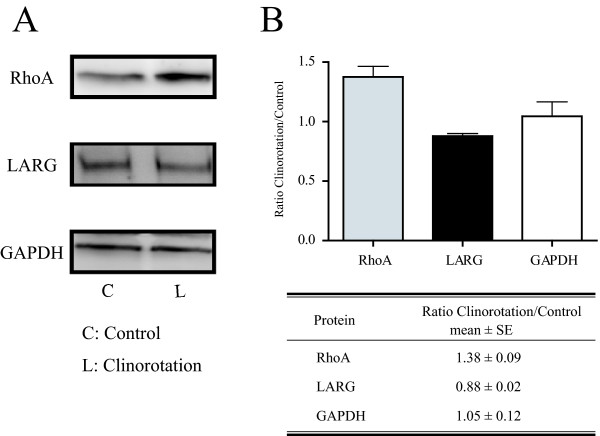
**Immunoblotting of LARG and RhoA**. The LARG expression level was decreased under altered gravity, whereas the expression level of RhoA was higher in the clinorotated cells than in the control cells. Cell lysis and immunocomplex detection of LARG, RhoA, and GAPDH were performed as described in the Methods section. In panel A, lanes C and L indicate the lysates prepared from the stationary control cells and the clinorotated cells, respectively. Each lysate contains 10 μg of total proteins. Panel B shows the density of the western blots in panel A quantified by the Quantity One software (Bio-Rad). The data shown are representative of three individual experiments. Values are represented as means ± SE (*p *< 0.05).

**Figure 5:**
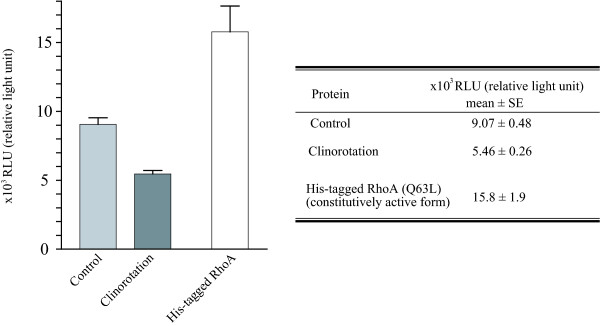
**Rho activity assay**. The active form of RhoA was luminometrically detected using a G-LISA kit. RhoA activity decreased significantly in the clinorotated cells. The lysate was extracted from cells that were cultured under 3D clinostat for 72 h. The lysate including 60 μg of total proteins was immediately reacted with a reaction reagent included in the kit. Values were obtained from eight separate experiments performed in triplicates and are represented as means ± SE (*p *< 0.05, control vs. clinorotation). His-RhoA (Q63L), a constitutively active RhoA protein containing a glutamine to leucine substitution at residue 63 was used as positive control in the G-LISA reaction.

Western blot was performed to examine the amount of LARG and RhoA proteins. As shown in Figure 4, the expression level of immunoreactive LARG densitometrically decreased to 87.8%; however, the RhoA expression level increased to 138% under clinorotation. Furthermore, the amount of activated RhoA was estimated using the G-LISA RhoA activation assay kit (see "Methods" section). As shown in Figure 5, the level of active RhoA in the clinorotated cells was 60.2% of that in the stationary control cells. These results indicated that the amounts of the LARG proteins and active RhoA decreased under simulated microgravity.

## Discussion

Previous reports demonstrated that the effects of microgravity on biological events vary. The microgravity environment has been reported to cause reduction in the secretion of growth hormones [22], blunting of the response to mitogen activation in T cells [23-25], retardation of osteoblast growth, and disorganization of cytoskeletal formation [26,27]. Cytoskeletal formation is highly sensitive to alteration in gravity. It was reported that in cells, the actin microfilament system constitutes the gravity-sensitive cell component [28] and that microgravity affects cytoskeletal formation [1,27]. This disorganization is caused by differences in the gravity level between the space (microgravity) and ground (1G) environments. In ground-based studies, a 3D clinostat is commonly used to simulate microgravity conditions. It has been reported that the results obtained using clinorotation were similar to those obtained in spaceflight experiments, suggesting that clinorotation is effective in simulating microgravity [2,9,29]. However, the mechanism by which the gravity signal is converted to an intercellular signal is unclear.

It is well known that the small G proteins of the Rho family regulate the remodeling of actin fibers [11,12,14]. Therefore, we hypothesized that the influence of microgravity on the Rho signaling pathway caused the disorganization of actin, and we examined the differences in gene expression between the static and clinorotated cells by using the mRNA fingerprinting method. We found that several gene fragments responded to the altered gravity. Subsequently, we identified a part of the LARG fragment that was downregulated under clinorotation. The *LARG *gene has been identified in some vertebrates [20,30]; however, this is the first study that has identified the entire bovine *LARG *gene. Motif analysis of the bovine LARG protein sequence demonstrated the presence of four conserved functional domains – the DH, PH, PDZ, and RGS domains. These four domains displayed a high degree of sequence conservation in the bovine and human/mouse proteins.

Our western blotting result showed that the total amount of Rho protein increased in the clinorotated cells, although the disorganization of actin fibers was observed. These results indicated that clinorotation affects not only Rho expression but also Rho activation. The real-time quantitative PCR and western blotting results demonstrated that the LARG expression significantly decreased in the clinorotated cells (Figures 3 and 4). In mammalian cells, three RhoGEFs, namely, LARG, p115-RhoGEF, and PDZ-RhoGEF have been identified as RhoA-selective RhoGEFs [31-33]. These RhoGEFs have close homologs and contain a regulator of G protein homology domains. In our results, the level of the LARG proteins showed a milder decrease (87.8% of the level in the stationary control) than that of active RhoA (60.2% of the normal level). We suspected downregulated expression of not only the LARG proteins but also the other RhoGEFs. In consequence, the downregulation of these RhoGEFs does not induce Rho activation and subsequently results in the disorganization of the actin fibers under simulated microgravity.

**Figure 6:**
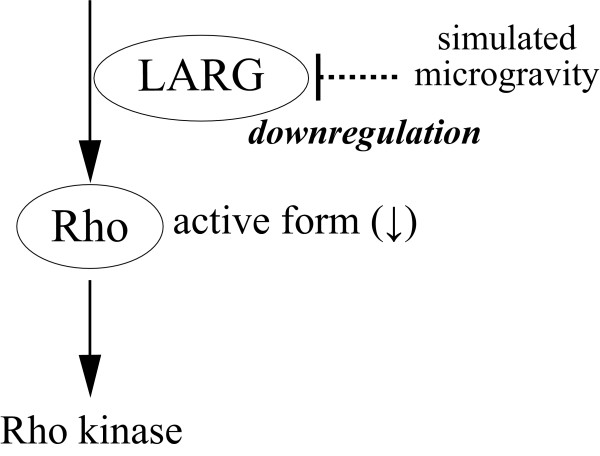
**Proposed regulation of Rho signaling**. The Rho signaling pathway may play an important role in the mechanism of gravity sensation in cells. Our results indicate that the gene expression of RhoGEF (LARG) is downregulated under simulated microgravity. As a result of the downregulation, the activation level of Rho becomes lower and the actin fibers are subsequently disorganized. The upstream and/or downstream regulation of Rho signaling may be involved in the process that converts the gravity signal into the intercellular signal.

We demonstrated that clinorotation induces the inactivation of Rho; however, the manner in which LARG transcription is downregulated under simulated microgravity has yet to be elucidated. Furthermore, as shown in Figure 6, we assumed that changes in the level of gravity also affect the upstream regulation of Rho, such as that via the heterotrimeric G proteins [14], or the downstream regulation of Rho via Rho effectors such as p160ROCK (Rho kinase) [34,35]. Recently, it has been pointed out that numerous cytoskeletal and signaling molecules mediate the interaction between actin and the focal adhesion molecules involved in mechanosensitivity [36]. In order to identify the exact molecule that converts the gravity signal into the intercellular signal, it is necessary that the upstream and downstream regulation of Rho signaling, including regulation via heterotrimeric G proteins, RhoGEFs, and Rho effectors such as Rho kinase, be the focus of future investigations.

## Conclusion

We concluded that the simulated microgravity generated by clinorotation affected Rho activation, particularly, the *LARG *gene expression, and this effect resulted in the disorganization of the actin fibers. This conclusion supported the previous observations of spaceflight and ground-based experiments [2,9,29].

## Methods

### Cell culture

BBME cells and cell culture reagents, including the CS-C medium kit, were purchased from Cell Systems Corporation (Kirkland, WA). The BBME cells were maintained, as described in the manufacturer's instructions, at 37°C with 5% CO_2_/95% air and 100% relative humidity in the CS-C medium.

### 3D clinorotation

The day before clinorotation, 1 × 10^5 ^BBME cells were plated into OptiCells (BioCrystal, Westerville, OH). After 24 h, the medium was replaced with fresh medium and the plated cells were subjected to 3D clinorotation in an apparatus. They were rotated at 37°C on the 3D clinostat apparatus in a 5% CO_2 _incubator. The cycles of rotation were controlled by the computer at an outer frame rotation of 10 rpm and an inner frame rotation of 13 rpm to cancel the dynamic simulation of gravity in any direction. The OptiCells were continuously rotated for 72 h without changing the medium. The control cells were incubated in parallel under the same conditions; however, they were not subjected to clinorotation. For the observation of the fluorescence images of the cells, a Lab-Tek II chamber slide system (Nalge Nunc International, Napperville, IL) was used instead of the OptiCells for cell culturing.

### Immunofluorescence microscopy

After rotation for 72 h on the 3D clinostat apparatus, the cells plated on a Lab-Tek chamber slide were washed once with phosphate-buffered saline (PBS) and fixed with 4% formaldehyde in PBS for 10 min. The fixed cells were washed twice with PBS and permeabilized with 0.5% Triton X-100 in PBS for 5 min. Subsequently, the cells were incubated with PBS containing 4% rhodamine-phalloidin (Invitrogen, Carlsbad, CA) and 20 ng/μl anti-paxillin antibody (Upstate, Lake Placid, NY) in combination with Alexa Fluor 488 (Molecular Probes, Eugene, OR). The cells were sufficiently rinsed in water and were then washed three times with PBS. Images were generated using a Leica Q550FW fluorescence imaging system (Leica Microsystems, Wetzlar, Germany).

### Isolation of total RNA, RT-PCR-based differential display, and isolation of differentially expressed gene segments

Total RNA was isolated from the clinorotated and stationary control cells by using Isogen, and first-strand cDNA synthesis followed by PCR were performed using an mRNA fingerprinting kit (Nippon Gene, Tokyo, Japan). The sets of primers used for this reaction are shown in the additional files (see Additional file Table 1). The cDNA samples obtained from the clinorotated and stationary control cells were electrophoresed on a 4% polyacrylamide gel and stained with SYBR Green I (Invitrogen). A differentially expressed band was excised from the acrylamide gel, and DNAs were extracted from the gel slice by using SUPREC-01 (TaKaRa Bio Inc., Shiga, Japan). The extracted cDNA fragments were further reamplified five times, and the significantly amplified fragments were cloned into a TA cloning vector by using pGEM-T Easy Vector System (Promega, Madison, WI). The cloned DNA sequences were determined by an ABI Prism 310 genetic analyzer (Applied Biosystems, Foster City, CA). The kits were used according to the manufacturer's instructions.

### Identification of bovine LARG cDNA

RT-PCR was carried out using an LA PCR in vitro cloning kit (TaKaRa Bio). 5' and 3' RACE procedures were performed using the 5'- and 3'-full RACE Core Sets, respectively (TaKaRa Bio). The primer sets of each procedure mentioned above were used as shown in the additional files (see Additional file Table 2). All procedures were performed according to the manufacturer's instructions. The primers were designed using GENETYX software version 6 (Genetyx Corporation, Tokyo, Japan).

### Real-time quantitative PCR

The expression levels of LARG and β-actin mRNA were measured using the real-time quantitative PCR method. The following primer pairs were used for LARG and β-actin, respectively: 5'-TGCTCACACCAGCTCCAGAAG-3' and 5'-CCTAGAGCAGGCAGTTACCAACAC-3' and 5'-GATGTGGATCAGCAAGCAGGAGTA-3' and 5'-AAGCATTTGCGGTGGACGA-3'. The reaction mixture was prepared using an SYBR RT-PCR kit (TaKaRa Bio). The amplification reaction was performed according to the manufacturer's instructions. Reaction and fluorescence monitoring were carried out by using a Smart Cycler real-time PCR system with Smart Cycler software version 2.0C (Cepheid, Sunnyvale, CA).

### Immunoblot analysis

After clinorotation for 72 h, the cells were washed twice with PBS. The whole cell lysate from the cells was prepared using a lysis buffer containing 25 mM Tris-HCl, pH 7.5; 150 mM NaCl; 5 mM MgCl_2_; 1% NP-40; 1 mM dithiothreitol; and 5% glycerol at 4°C. The whole cell extract was subjected to sodium dodecyl sulfate polyacrylamide gel electrophoresis (SDS-PAGE; 5–10% gradient gel for LARG detection and 15% gel for RhoA and GAPDH detection) and transferred to a polyvinylidene difluoride membrane (Hybond-P; Amersham Biosciences, Piscataway, NJ) at 15 V for 60 min at room temperature. The membrane was blocked with 5% dry fat milk and incubated subsequently with Tris-buffered saline containing 0.05% Tween 20 with a 1:100 dilution of mouse monoclonal anti-RhoA antibody (26C4; Santa Cruz Biotechnology, Inc., Santa Cruz, CA), a 1:100 dilution of rabbit polyclonal anti-LARG antibody (H70; Santa Cruz), and a mouse monoclonal anti-glyceraldehyde-3-phosphate (GAPDH) antibody (Imgenex, San Diego, CA). Immunocomplexes were visualized using an ECL Plus chemiluminescence kit (Amersham). Signals were densitometrically quantified using Quantity One software (Bio-Rad Laboratories Inc., Hercules, CA).

### Rho GTPase activity assay

Active RhoA was measured using a G-LISA RhoA activation assay biochem kit (luminnometric assay; Cytoskeleton, Inc., Denver, CO) according to the manufacturer's instructions. The chemiluminescence signals were detected using a Powerscan HT multidetection microplate reader (Dainippon Pharmaceutical, Osaka, Japan).

### Statistics

Statistical analysis was performed using the Student's *t*-test by PRISM software (GraphPad software Inc., San Diego, CA). Significance was accepted at *p *< 0.05. All experiments were repeated at least three times. Values are expressed as mean ± SE.

## Abbreviations

aa: amino acid residues

ALCAM: human cell adhesion factor

BBME: bovine brain microvascular endothelial

bp: base pairs

DH: Dbl homology

EST: expressed sequence tag

GAPDH: glyceraldehyde-3-phosphate dehydrogenase

GEF: guanine nucleotide exchange factor

LARG: leukemia-associated Rho GEF factor

MLL: mixed-lineage leukemia

ORF: open reading frame

PCR: polymerase chain reaction

PDZ: domain present in PSD-95, Dlg and ZO-1/2;

PH: pleckstrin homology

RACE: rapid amplification of cDNA ends

RGS: regulator of G protein signaling

RT-PCR: reverse transcription-polymerase chain reaction

## Authors' contributions

AH drafted the manuscript, performed the western blotting studies, and participated in the design of the study. MI and TY participated in the sequence alignment of bovine LARG. MS participated in the Rho GTPase activity assay and quantitative real-time PCR analyses. NI supervised the study. All authors read and approved the final manuscript.

## Supplementary Material

Additional File 1 Table 1. Sequences of primers for mRNA fingerprinting differential display Click here for file
