# Supplementary material for: Influence of simulated microgravity on the activation of the small GTPase Rho involved in cytoskeletal formation – molecular cloning and sequencing of bovine leukemia-associated guanine nucleotide exchange factor
Source: BMC Biochem. 2006 Jun 28;7:19. doi: 10.1186/1471-2091-7-19 (PMC1524780; doi:10.1186/1471-2091-7-19)
Supplement: Additional File 1 — Table 1. Sequences of primers for mRNA fingerprinting differential display [file 1471-2091-7-19-S1.doc]

**Additional Files**

Table 1. Sequences of primers for mRNA fingerprinting differential display

| 5′ arbitrary primers |  |
| --- | --- |
| AP-A-02 | 5′-TGGATTGGTC-3′ |
| AP-A-03 | 5′-CTTTCTACCC-3′ |
| AP-A-04 | 5′-TTTTGGCTCC-3′ |
| AP-A-05 | 5′-GGAACCAATC-3′ |
| AP-A-08 | 5′-TGGTAAAGGG-3′ |
| AP-A-14 | 5′-GTACAAGTCC-3′ |
| AP-A-17 | 5′-GATCTGACAC-3 |
| AP-A-18 | 5′-GATCTCAGAC-3′ |
| AP-A-23 | 5′-GATCTGACTG-3′ |
| AP-A-24 | 5′-GATCATGGTC-3′ |
| AP-B-04 | 5′-CGTCTTTCTG-3′ |
| AP-B-05 | 5′-TACTGTTGCC-3′ |
| AP-B-09 | 5′-ATCCTCTTCC-3′ |
| AP-B-07 | 5′-CTGTCATCAG-3′ |
| AP-B-10 | 5′-ACTGAGATCC-3′ |
| AP-B-15 | 5′-CGAAAACCAG-3′ |
| AP-B-16 | 5′-GGATTCACTG-3′ |
| AP-B-17 | 5′-ACTTTTGGCG-3′ |
| AP-B-21 | 5′-CATTCACCTC-3′ |
| AP-B-25 | 5′-CTTCAGTTCC-3′ |
| 3′ anchor primers |  |
| GT15A | 5′-GTTTTTTTTTTTTTTTA-3′ |
| GT15C | 5′-GTTTTTTTTTTTTTTTC-3′ |
| GT15T | 5′-GTTTTTTTTTTTTTTTT-3′ |

Table 2. Sequence of primers for molecular cloning and sequence of bovine LARG

| LARG-1F | 5′-GGCCCCAATGAGTGGCACACAGTCTAC-3′ |
| --- | --- |
| LARG-1R | 5′-GCAGCTGAGGAATGTGTTTCTTGGCCTCTT-3′ |
| LARG-2F | 5′-GCTGCAAGAGCAGTTATCCAAAGCCA-3′ |
| LARG-2R | 5′-AGGCTTTAGTCCCAATAACACTTCTCGACT-3′ |
| LARG-3F | 5′-TAGATGGCACACCTCGTACT-3′ |
| LARG-3R | 5′-TTATCTGTTGCCACTTGTCGAA-3′ |
| LARG-4F | 5′-CCTCTAATCTGAAGTTGTCAGAATACCC-3′ |
| LARG-4R | 5′-GGATCAAATAGTTTCCTGAGATGCGT-3′ |
| LARG-5F | 5′-CTCCACGGGATTCAGTAGTACTGGCATTC-3′ |
| LARG-5R | 5′-TGAGGAAAGAATAATTACGCA-3′ |
| LARG-6F | 5′-AATTAAGCAGGACCTTCCAGT-3′ |
| LARG-6R | 5′-GTACATATTGTCGTTAGAACGCGTAATACGACTCA-3′  (*Pst I* cassette primer including PCR in vitro cloning kit) |
| LARG-7F | 5′-GATTGGGACAGCTTAATGACCTC-3′ |
| LARG-7R | 5′-CTGATCTAGAGGTACCGGATCC-3′  (including 3′Full RACE Core Set 3sites adaptor primer) |
| RC5-RTP | 5′-ACCTGTCTGTACTCC-3′ |
| RC5-S1-F | 5′-GAAGGATGACAATGGATTTGGG-3′ |
| RC5-S1-R | 5′-CTTCTTGGAGGAGCTATCTGTG-3′ |
| RC5-S2-F | 5′-CAGTCTGTCAAAGAAGATGG-3′ |
| RC5-S2-R | 5′-CTGCTTCTTATCTGTTGGTG-3′ |
